# A Lumped-Parameter Model of the Cardiovascular System Response for Evaluating Automated Fluid Resuscitation Systems

**DOI:** 10.1109/access.2024.3395008

**Published:** 2024-05-08

**Authors:** YEKANTH RAM CHALUMURI, GHAZAL ARABIDARREHDOR, ALI TIVAY, CATHERINE M. SAMPSON, MUZNA KHAN, MICHAEL KINSKY, GEORGE C. KRAMER, JIN-OH HAHN, CHRISTOPHER G. SCULLY, RAMIN BIGHAMIAN

**Affiliations:** 1 Department of Mechanical Engineering, University of Maryland, College Park, MD 20742, USA; 2 Department of Anesthesiology, The University of Texas Medical Branch, Galveston, TX 77555, USA; 3 Office of Science and Engineering Laboratories, Center for Devices and Radiological Health, United States Food and Drug Administration, Silver Spring, MD 20993, USA

**Keywords:** Cardiovascular system, fluid perturbation, mathematical model credibility assessment, in silico evaluation of physiological closed-loop control medical devices

## Abstract

Physiological closed-loop controlled (PCLC) medical devices, such as those designed for blood pressure regulation, can be tested for safety and efficacy in real-world clinical settings. However, relying solely on limited animal and clinical studies may not capture the diverse range of physiological conditions. Credible mathematical models can complement these studies by allowing the testing of the device against simulated patient scenarios. This research involves the development and validation of a low-order lumped-parameter mathematical model of the cardiovascular system’s response to fluid perturbation. The model takes rates of hemorrhage and fluid infusion as inputs and provides hematocrit and blood volume, heart rate, stroke volume, cardiac output and mean arterial blood pressure as outputs. The model was calibrated using data from 27 sheep subjects, and its predictive capability was evaluated through a leave-one-out cross-validation procedure, followed by independent validation using 12 swine subjects. Our findings showed small model calibration error against the training dataset, with the normalized root-mean-square error (NRMSE) less than 10% across all variables. The mathematical model and virtual patient cohort generation tool demonstrated a high level of predictive capability and successfully generated a sufficient number of subjects that closely resembled the test dataset. The average NRMSE for the best virtual subject, across two distinct samples of virtual subjects, was below 12.7% and 11.9% for the leave-one-out cross-validation and independent validation dataset. These findings suggest that the model and virtual cohort generator are suitable for simulating patient populations under fluid perturbation, indicating their potential value in PCLC medical device evaluation.

## I. INTRODUCTION

Physiological closed-loop controlled (PCLC) medical devices offer potential valuable support for healthcare professionals working in high-pressure clinical settings where frequent adjustments to medication are necessary to achieve a desired physiological target [1], [2], [3], [4]. These devices can provide real-time monitoring and adjustment of medication dosages based on the patient’s physiological responses, thereby reducing the workload for physicians and nurses while ensuring optimal patient care, as emphasized in 2015 public workshop on PCLC challenges and opportunities held by the U.S. Food and Drug Administration (FDA) [5]. However, there are concerns that relying solely on these devices may create a false sense of security for caregivers, potentially leading to unsafe practices [1], [6]. Therefore, it is crucial to recognize the limitations of PCLC devices and determine the conditions under which they may not function safely. The limitations of PCLC devices can manifest in various ways, including issues related to human interaction with the device, interoperability between the closed-loop controller and internal/external sensors and actuators, and the ability of the PCLC to respond appropriately to different physiological conditions and external disturbances [1], [7].

Using data solely from limited animal and clinical studies may not offer a sufficiently comprehensive testing platform for the PCLC devices [8], [9], [10]. It is recommended to test these devices under different operating conditions and physiological scenarios that may occur throughout their lifetimes. However, since limited animal and clinical studies may not cover all realistic scenarios that these devices may encounter, it may be necessary to employ additional testing methods such as simulations and modeling to more thoroughly evaluate the device [11], [12], [13], [14], [15], [16]. A credible mathematical model and virtual cohort generation tool can effectively generate a diverse range of possible operating conditions and scenarios [8], [9], [17]. These virtual patients can serve as a valuable complement to animal and clinical studies, enabling researchers to ensure the safety and effectiveness of PCLC devices before they are used in practice. By utilizing these mathematical models, researchers can simulate various patient characteristics, boundary conditions, and physiological scenarios to identify potential risks and unsafe conditions that may compromise patient outcomes.

PCLC medical devices have significant potential in hemodynamic management and fluid resuscitation, making this area a major application of interest [18], [19], [20]. This emphasizes the critical need for credible mathematical models of the cardiovascular system, particularly in the context of hemorrhage and fluid infusion, i.e., fluid perturbation. Although previous studies have attempted to develop such models [17], [21], [22], [23], none have yet been validated for their predictive capability performance. In other words, simulated subjects generated based on training data were not employed to assess the replicability of test data. In addition, some of these models consist of hundreds of parameters, making it challenging to generate fully subject-specific virtual patients [21], [22], [23]. Many of these parameters are sourced from literature and cannot be inferred from commonly measured physiological variables [22]. Moreover, the large number of parameters in these models poses difficulties when working with virtual patient cohort generation tools. Various physiological scenarios can be mimicked using different combinations of model parameters. Retaining certain parameters while generating virtual subjects may result in the failure to reach different physiological states. Conversely, attempting to vary all parameters can be computationally demanding and may require millions of simulations to replicate all possible scenarios, if such replication is even feasible.

To ensure the accuracy of in silico evaluation of PCLC medical devices, it is essential to address uncertainties related to the fidelity of mathematical models. This involves addressing any ambiguities surrounding the selection of appropriate performance measures for model validation to ensure that the models are reliable and capable of accurately predicting PCLC medical device performance in real-world scenarios. Most cardiovascular mathematical models in the past were evaluated solely at or near their nominal response, with qualitative assessments of their physiological response or only, their calibration, i.e., fitting, performance (e.g., [21], [22], [23]). Nonetheless, these evaluations do not strongly support the criteria for credibility evidence [24], [25]. In addition, it is crucial that PCLC medical devices perform as expected under different boundary conditions such as varying rates of hemorrhage and fluid infusion, or when interoperating with other devices. Therefore, it is important to validate the mathematical models against independent test datasets that subject the models to various boundary conditions. This will help ensure that the models can accurately predict the performance of PCLC medical devices in different situations. Historically, models have been assessed on their ability to effectively calibrate physiological variables using training data, assuming that the PCLC device will be used in settings comparable to those used during calibration (e.g., [17]).

The objective of this study is to develop a comprehensive mathematical model of the cardiovascular system using physiological first principles. The model is a low-order lumped parameter model, designed for use with virtual cohort generation tools. It takes rates of hemorrhage and fluid infusion as inputs and produces outputs for hematocrit (HCT), blood volume (BV), heart rate (HR), stroke volume (SV), cardiac output (CO), and mean arterial blood pressure (BP). We modified our previously developed compartment method for generating virtual subjects to ensure compatibility with the specifications of this new mathematical model. Performance metrics for predictive capability have been adapted and expanded from our recent work [8]. The model’s calibration was achieved using data from 27 sheep subjects, and its predictive capability was evaluated through a leave-one-out cross-validation procedure. Additionally, we assessed the model’s credibility using 12 independent swine test subjects that underwent a different fluid perturbation protocol. The model can be employed to generate simulated patients for designing or evaluating PCLC medical devices operating in the presence of fluid perturbations.

## II. MATERIALS AND METHODS

This section introduces a novel mathematical model of the cardiovascular system, featuring sub-models for HCT, HR, SV (CO), and BP. The experimental data used for model calibration as well as model predictive capability assessment are presented. We also outline the methods used for parameter estimation, model calibration assessment, and evaluating the model’s predictive performance. The software code for the mathematical model and methods employed in assessing predictive capability performance, along with instructions for its usage, is provided in the Appendix.

### A. MATHEMATICAL MODEL OF THE CARDIOVASCULAR SYSTEM RESPONSE TO FLUID PERTURBATION

The mathematical model of the cardiovascular system, along with its corresponding sub-models is provided. To ensure clarity and cohesiveness, each sub-model is presented in a dedicated sub-section, despite the sub-models being all connected and integrated.

#### 1) SUB-MODEL 1: BLOOD VOLUME

**FIGURE 1.**
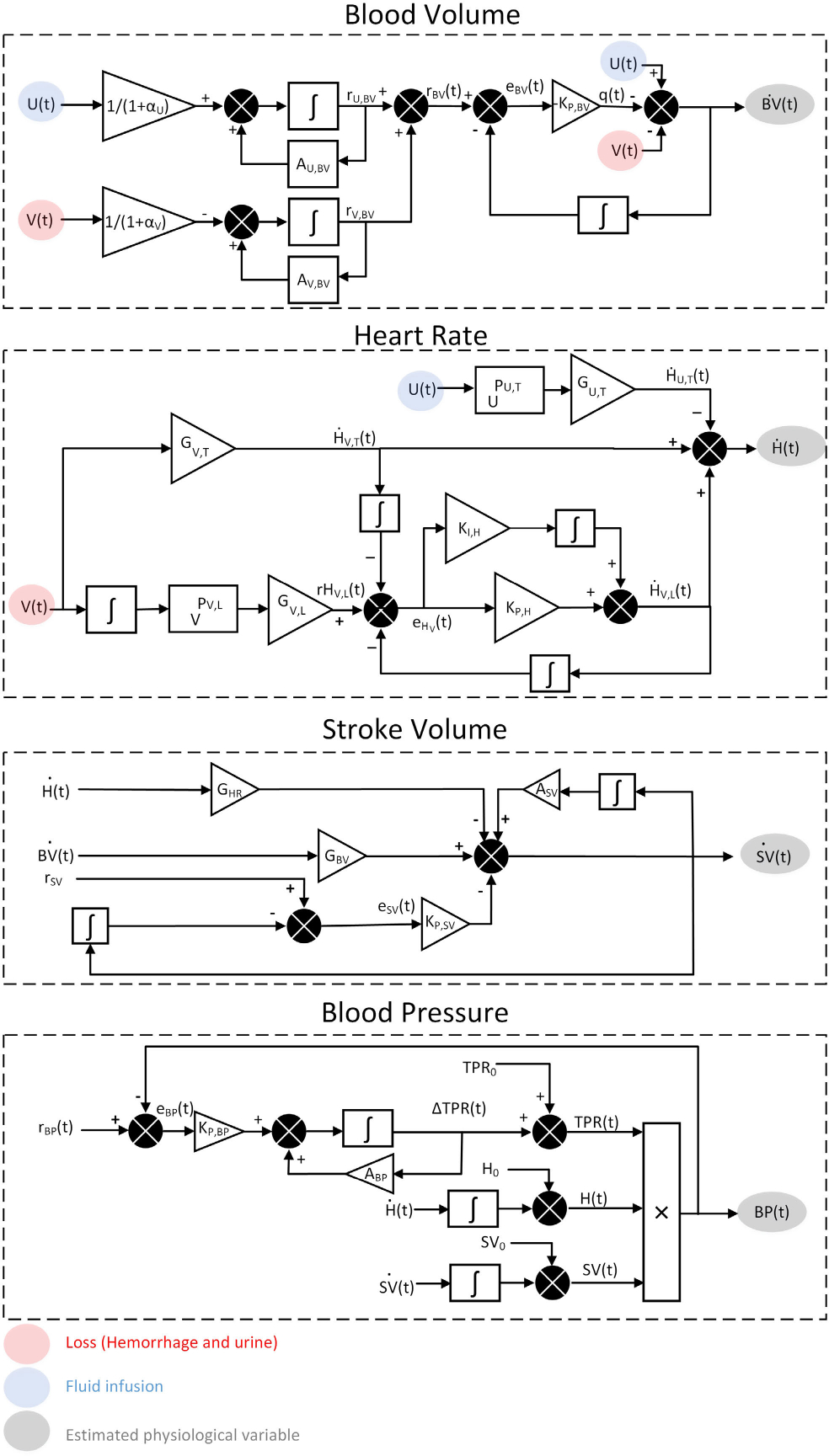
Mathematical model of the cardiovascular system response to fluid perturbation. The mathematical model receives rate of loss (hemorrhage and urine) and fluid infusion, and outputs blood volume, heart rate, stroke volume, cardiac output, and blood pressure. For simplicity, the mathematical model of each physiological variable is presented separately, although they are all interrelated, with the output of one sub-model serving as an input to another sub-model.

The full details of the mathematical model of BV can be found in our previous research [9], [26], [27]. The model receives the rate of fluid infusion U(t)>0, in liters per minute (l/m) and rate of loss V(t)>0(1/m), where V(t) includes both hemorrhage and urine. The model operates on a two-compartment system, comprising intravascular and interstitial spaces, and relies on a feedback-control mechanism that governs fluid transfer between them, thereby maintaining their mass balance (Figure 1). By incorporating two constant distribution ratios $\alpha_{u}$ and $\alpha_{v}$ for fluid infusion and loss, the model determines a subject-specific reference change in BV over time based on the given inputs:

(1) $${\dot{r}}_{U,BV}(t)=A_{U,BV}r_{U,BV}(t)\;\;\;+\frac{1}{1+\alpha_{U}}U(t),r_{U,BV}(0)=0$$

(2) $${\dot{r}}_{V,BV}(t)=A_{V,BV}r_{V,BV}(t)\;\;\;-\frac{1}{1+\alpha_{V}}V(t),r_{V,BV}(0)=0$$

where $A_{i,BV}(1/m)$ and $r_{i,BV}(t),i\in \left\{U,V\right\}$ (1) denote the state parameter and reference steady-state change in BV due to infusion or loss. The reference change in BV is calculated as the sum of the reference steady-state change under infusion and loss:

(3) $$r_{BV}(t)=r_{U,BV}(t)+r_{V,BV}(t)$$

Once the reference change in BV is obtained, a proportional controller [28] is used to mimic the rate of fluid shift between the intravascular and interstitial spaces, q(t)(1/m), resulting in the desired change in BV (1):

(4) $$\dot{BV}(t)=U(t)-V(t)-q(t)$$

(5) $$q(t)=K_{P,BV}\left(BV(t)-BV(0)-r_{BV}(t)\right)$$

where $K_{P,BV}(1/m)$ is the controller gain. It is noted that a simpler version of the refined model in [9] is used here, where the integral controller is removed from the fluid shift mechanism. Once BV is obtained, hematocrit (HCT) (%), the volume percentage of red blood cells (RBC) (l) in blood, is modeled:

(6) $$HCT(t)=\frac{RBC(t)}{BV(t)}$$

RBC is computed via the amount of RBC loss due to hemorrhage:

(7) $$RBC\left(t\right)=HCT_{0}BV_{0}-\int_{0}^{t}V\left(l\right)HCT\left(l\right)dl$$

where $HCT_{0}$ and $BV_{0}$ are baseline HCT and BV, respectively. The model has 7 parameters, including $\left\{\alpha_{U},\alpha_{V},A_{U,BV},A_{V,BV},K_{P,BV}\right\}$ as well as two baseline parameters $\left\{HCT_{0},\;BV_{0}\right\}$. Detailed model credibility assessment activities for this sub-model can be found in [9].

#### 2) SUB-MODEL 2: HEART RATE

The full details of the validated HR mathematical model can be found in our previous research [8]. This mathematical model is based on the physiological response of HR to fluid infusion and loss, and is designed to reflect the corresponding short-term and long-term physiological behavior. After experiencing a blood loss, a rise in HR (bpm) occurs due to the increase in sympathetic nervous system activity. This initial short-term increase is then followed by a long-term increase, which is caused by a decrease in arterial BV and lower BP. In contrast, administering fluid infusion causes a temporary decrease in HR, which helps to alleviate some of the effects of hemorrhage. The model takes rates of infusion and loss and outputs HR response to fluid perturbation.

The model replicates the above mentioned short- and long-term behavior using the following relationships. First, for a given loss rate V(t), a proportional transient increase in HR is obtained via a constant gain of $G_{V,T}(1/lm)$ (Figure 1):

(8) $${\dot{H}}_{V,T}(t)=G_{V,T}V(t)$$

In the same way as the BV model, the long-term increase in HR is modeled through a feedback-control mechanism with the following subject-specific reference long-term change in HR:

(9) $$rH_{V,L}\left(t\right)=\int_{0}^{t}G_{V,L}V{\left(l\right)}^{P_{V,L}}dl$$

where $G_{V,L}(1/lm)$ and $P_{V,L}(-)$ are subject-specific constant gain and power terms, respectively. In addition, a feedback control system calculates the long-term increase in HR as a function of time, denoted as $H_{V,L}(t)$, by analyzing the short- and long-term changes in HR caused by hemorrhage and urine, and comparing them to the reference $rH_{V,L}(t)$.

(10) $${\dot{H}}_{V,L}\left(t\right)=K_{P,H}e_{H_{V}}\left(t\right)+K_{I,H}\int_{0}^{t}e_{H_{V}}\left(l\right)dl$$

(11) $$e_{H_{V}}(t)=rH_{V,L}(t)-H_{V,T}(t)-H_{V,L}(t)$$

where $K_{P,H}(1/m)$ and $K_{I,H}\left(1/m^{2}\right)$ are the proportional and integral gains associated with the controller. Lastly, the amount of transient drop in HR due to fluid infusion, denoted as $H_{U,T}(t)$, is modeled through a nonlinear algebraic equation:

(12) $${\dot{H}}_{U,T}(t)=G_{U,T}U(t{)}^{P_{U,T}}dt$$

where $G_{U,T}(1/lm)$ and $P_{U,T}(-)$ are subject-specific constant gain and power terms for the infusion response, respectively. The rate of change in HR is the sum of short- and long-term responses to fluid perturbation:

(13) $$\dot{H}(t)={\dot{H}}_{V,L}(t)+{\dot{H}}_{V,T}-{\dot{H}}_{U,T}(t),H(0)=H_{0}$$

The model has 8 parameters, including:

$\left\{G_{V,T},G_{U,T},G_{V,L},P_{V,L},P_{U,T},K_{P,H},K_{I,H}\right\}$ as well as the baseline parameter $\left\{H_{0}\right\}$.

#### 3) SUB-MODEL 3: STROKE VOLUME

The model takes into account the physiological factors that affect SV (Figure 1). One important factor is HR, which is inversely proportional to SV. When the heart beats faster, there is less time for the ventricles to fill with blood before they contract again. This can lead to a decrease in the amount of blood that is pumped out with each beat, resulting in a smaller SV(l):

(14) $${\dot{SV}}_{H}(t)=-G_{HR}\dot{H}(t)$$

The parameter $G_{HR}\;(1.m)$ denotes the gain factor that maps the rate of change in HR to SV. The model also incorporates a proportional relationship between SV and BV, where SV increases with BV through the gain factor $G_{BV}(-)$:

(15) $${\dot{SV}}_{BV}(t)=G_{BV}\dot{BV}(t)$$

And lastly, the model accounts for a subject-specific reference level of SV, whereby each individual has a target steady-state SV level $r_{SV}\;(l)$ that can vary among subjects in response to changes in heart contractility. Changes in SV resulting from perturbations in HR and BV can trigger a feedback control system, which works to regulate SV back toward the reference value:

(16) $${\dot{SV}}_{r}(t)=K_{P,SV}\left(r_{SV}-SV(t)\right)$$

where $K_{P,SV}(1/m)$ is the proportional control gain. To account for the dynamics of SV changes over time, the SV model includes a state parameter $A_{SV}(1/m)$:

(17) $$\dot{SV}(t)=A_{SV}\left(SV(t)-SV_{0}\right)+S{\dot{V}}_{BV}(t)\;\;\;+S{\dot{V}}_{r}(t)-S{\dot{V}}_{H}(t),SV(0)=SV_{0}$$

The model has 6 parameters, including:

$\left\{G_{HR},G_{BV},K_{P,SV},r_{SV},A_{SV}\right\}$ as well as the baseline parameter $\left\{SV_{0}\right\}$.

#### 4) SUB-MODEL 4: BLOOD PRESSURE

The model of BP is based on a control-oriented model of total peripheral resistance (TPR). TPR (mmHg.m/l) plays a crucial role in regulating BP, as it reflects the resistance to blood flow in the peripheral vasculature. The model incorporates a subject-specific reference level for BP, where deviations from this reference level can trigger adjustments in TPR to bring BP back toward the reference value via the state parameter $A_{BP}(1/m)$ and the proportional controller gain $K_{P,BP}(1/1)$ (Figure 1).

(18) $$T\dot{P}R(t)=A_{BP}\left(TPR(t)-TPR_{0}\right)\;\;\;+K_{P,BP}\left(r_{BP}-BP(t)\right),TPR(0)=TPR_{0}$$

(19) BP(t)=H(t)SV(t)TPR(t)=CO(t)TPR(t)

where CO(t) denotes CO(1/m) and $TPR_{0}$ is the baseline TPR. The model has 4 parameters, including $\left\{r_{BP},K_{P,BP},A_{BP}\right\}$ as well as the baseline parameter $\left\{TPR_{0}\right\}$.

Although the sub-models were presented separately, it is important to note that the model is an integrated system, wherein all sub-models interact collaboratively to produce the overall model outputs. For example, simulated BV and HR variables are inputs to the SV sub-model, and simulated HR and SV variables are inputs to the BP sub-model, as shown in Figure 1.

### B. EXPERIMENTAL DATA

This study utilized two distinct sets of data. Both data collection protocols were approved by the Institutional Animal Care and Use Committee (IACUC) at the University of Texas Medical Branch. The first dataset was utilized for model development and an initial round of model assessment through a leave-one-out cross-validation procedure. The second dataset served as independent test data for the subsequent validation of the model.

#### 1) SHEEP DATA

This dataset includes 27 individual datasets collected from 22 sheep subjects that underwent conscious hemorrhage and fluid resuscitation. Full details about this dataset can be found in a prior study [29]. They were administered Lactated Ringer’s solution (LR), a commonly used crystalloid fluid. Five of the sheep subjects received Hextend (HEX) at a five-day interval within their LR study. The sheep subjects were awake throughout the experiment. Each experiment spanned over 3 hours, comprising a baseline measurement followed by a hemorrhage of 25 ml/kg for 15 minutes. The subjects were then infused with fluids for the last 150 minutes using a closed-loop controller, as described in [29]. Two additional hemorrhages of 5 ml/kg were administered at 50 and 70 minutes after the start of experiment, each lasting five minutes. ${\mathrm{BV}}_{0}$ was measured in each animal using indocyanine green dye (ICG) [30]. The physiological variables, including HCT, HR, CO, and BP, were measured every 5 or 10 min throughout the experiment.

#### 2) SWINE DATA

This dataset consists of 12 individual datasets collected from 12 swine subjects who underwent general anesthesia followed by hemorrhage and fluid resuscitation. Female Yorkshire pigs, aged 3–4 months and weighing 35 ± 7 kg, were acquired from a USDA-licensed supplier (USDA license 74-R-065; U.T. M.D. Anderson D.V.S., Bastrop, TX). Following a 15-day quarantine, a veterinary examination confirmed their health status. Prior to the experiment, the pigs underwent an overnight fast and received intramuscular injections of 1.2 to 2.8 mg/kg ketamine (KetaVed; Vedco Inc, St Joseph, MO), 1.2 to 2.8 mg/kg xylazine (AnaSed; Akorn, Decatur, IL), 2.3 to 5.7 mg/kg Terazol (Zoetis Inc, Kalamazoo, MI), and 0.3 mg buprenorphine (Par Pharmaceutical Cos. Inc, Spring Valley, NY). General anesthesia was maintained with an intravenous infusion of 80 to 150 *μ*g/kg/min of propofol (Diprivan; Fresenius Kabi USA, LLC, Lake Zurich, IL). Following endotracheal intubation, the pigs were mechanically ventilated (Hamilton G5; Hamilton Medical AG, Bonaduz, Switzerland) with a tidal volume of 10–12 ml/kg, a respiratory rate of 12 to 15 breaths per minute, and a FiO2 of 0.5. Adjustments were made to the tidal volume and respiratory rate to maintain an end-tidal CO2 of 35 to 45 mmHg. Catheterization of femoral arteries and veins facilitated BP monitoring and fluid administration. Swan-Ganz and central venous catheterizations were performed on the right internal and external jugular veins, respectively. A splenectomy was conducted to eliminate potential splenic contraction and autotransfusion. During surgical preparation, PlasmaLyte A (Baxter, Deerfield, IL) was administered at a rate of 12 ml/kg/h. To prevent hypothermia, the intravenous fluid was prewarmed, and a warming blanket was applied. Comprehensive clinical monitoring, including temperature, electrocardiography, arterial BP (Philips HP Merlin), pulse oximetry (Masimo Radical-7; Masimo, Irvine, CA), and capnography (Datex-Ohmeda Capnomac Ultima), was implemented to ensure animal stability throughout the study. Euthanasia was performed with an intravenous infusion of 25 mg/kg ketamine followed by 1 to 2 mEq/kg of saturated KCl (Hospira Inc, Lake Forest, IL) upon completion of the study protocol. After 45 min of baseline measurement, subjects underwent a hemorrhage at a rate of body weight per minute until a BP of 40 mmHg was reached, followed by 500 ml per 70 kg boluses of 5% albumin, each succeeded by a 60 min washout. In some cases, subjects received an initial fluid infusion before the hemorrhage was applied. Each experiment lasted between 2 to 4 hours. The same physiological variables as the sheep subjects were measured every 5 or 10 min throughout the experiment. $BV_{0}$ was not measured in swine subjects, and was approximated by $BV_{0}=60\;ml/kg$ [31].

### C. MODEL PARAMETER ESTIMATION

We utilized data from sheep subjects to calibrate the model parameters, which comprises 25 parameters, of which 5 correspond to baseline values. To avoid estimation bias in the BV model parameters [9], [26], we included the measured $BV_{0}$ value for each subject. For simplicity, we also used the measured values for $HCT_{0}$ and $SV_{0}$ instead of identifying them, although it is worth noting that both parameters could be accurately estimated if included in the estimation procedure. The remaining 22 parameters were identified for each individual subject.

The maximum-likelihood estimation (MLE) was employed to identify the model parameters. MLE determines the parameter values that maximize the likelihood of the observed physiological variables, i.e., HCT, HR, SV and BP. By considering the error between the physiological data and their corresponding model outputs over time, denoted by $e\left(t_{k}\right)$, the physiological measurements can be presented as:

(20) $$v\left(t_{k}\right)=\overset{ˆ}{v}\left(t_{k}|\Theta \right)+e\left(t_{k}\right)$$

where k=1,…,K is the sequence of discrete observations, $\overset{ˆ}{v}\left(t_{k}|\Theta \right)$ is the model response at time $t_{k}$ for each variable v, and Θ represents the set of 22 model parameters. Assuming that $e\left(t_{k}\right)$ is distributed normally, independently, and identically, with a mean of $E\left(e\left(t_{k}\right)\right)=0$ and a variance of $V\left(e\left(t_{k}\right)\right)=\sigma^{2}$, the likelihood function of the error is presented as:

(21) $$N\left(e\left(t_{k}\right);0,\sigma^{2}\right)=\frac{1}{\sqrt{2\pi \sigma^{2}}}\;\;\;\times exp\left\{\frac{-1}{2\sigma^{2}}{\left(v\left(t_{k}\right)-\overset{ˆ}{v}\left(t_{k}|\Theta \right)\right)}^{2}\right\}$$

Thus, the likelihood function of Θ and $\sigma^{2}$ can be expressed as:

(22) $$L(\Theta ,\sigma )=\;\prod_{k=1}^{K}N\left(v\left(t_{k}\right);\overset{ˆ}{v}\left(t_{k}|\Theta ,\sigma^{2}\right)\right)\;\;={\left(2\pi \sigma^{2}\right)}^{-K/2}exp\left\{\frac{-1}{2\sigma^{2}}{\left(v\left(t_{k}\right)-\overset{ˆ}{v}\left(t_{k}|\Theta \right)\right)}^{\prime }\right.\;\;\left.\times \left(v\left(t_{k}\right)-\overset{ˆ}{v}\left(t_{k}|\Theta \right)\right)\right\}$$

By taking the logarithm of Eq. 22 the log-likelihood function $L^{*}$ is obtained:

(23) $$L^{*}(\Theta ,\sigma )=\;-\frac{K}{2}ln(2\pi )-\frac{K}{2}ln\left(\sigma^{2}\right)\;\;\;-\frac{1}{2\sigma^{2}}{\left(v\left(t_{k}\right)-\overset{ˆ}{v}\left(t_{k}|\Theta \right)\right)}^{\prime }\;\;\;\times \left(v\left(t_{k}\right)-\overset{ˆ}{v}\left(t_{k}|\Theta \right)\right)$$

Finally, the following optimization problem is used to identify the model parameters:

(24) $$\mu^{ML}\;=\left\{\Theta^{*},\sigma^{*}\right\}\;\;\;\;\;\;\;\;\;\;\;\;=arg\;\underset{\mu }{min}\left(-\sum_{i}L_{v_{i}}^{*}\left(\Theta ,\;\sigma \right)+2\beta {\left\|\Theta \right\|}_{2}\right)$$

where $v_{i}\in \{HCT,\;HR,\;SV,\;BP\}$, β>0 is the $L_{2}$ penalty term used to regularize the model, $∥.{∥}_{2}$ denotes the $L_{2}$ norm operator and penalizes the $L_{2}$ of the model parameters. This encourages the model parameters to be small and reduces the estimation variance, which helps to avoid overfitting. The solution to the optimization problem identifies model parameters that best replicate the physiological variables.

An iterative gradient-based optimization approach was implimented in MATLAB for the parameter estimation. For a rigorous exploration of the parameter space and to evaluate the uniqueness of the calibrated parameters, multiple optimization runs were conducted, each initiated from different random initial values. This helped ensure the reliability and robustness of the optimization results. Additionally, variance-based global sensitivity analyses were carried out for individual sub-models. These analyses involved assessing the impact of variations in the sub-model parameters on the overall model performance, considering both the model structure and the calibration data. The results confirmed the sensitivity of the parameters associated with each sub-model. The detailed analysis can be found in [8] and [9].

### D. MODEL ASSESSMENT

#### 1) MODEL CALIBRATION PERFORMANCE

The model was calibrated to each individual sheep subject and the model’s simulated variables HCT, HR, SV, CO, and BP were compared to their measured counterparts. The performance of the calibration was evaluated for each physiological variable using the normalized root-mean-square error (NRMSE) and normalized mean absolute error (NMAE):

(25) $$NRMSE_{v}=100\frac{\sqrt{\frac{\sum_{k=1}^{K}{\left(v\left(t_{k}\right)-\overset{ˆ}{v}\left(t_{k}\right)\right)}^{2}}{K}}}{\overset{‾}{v}}$$

(26) $$NMAE_{v}=100\frac{\frac{1}{K}\sum_{k=1}^{K}\left|v_{k}-{\overset{ˆ}{v}}_{k}\right|}{\overset{‾}{v}}$$

where $\overset{‾}{v}$ represents the mean value of the variable v in a subject. Lastly, the negative log-likelihood (-LL) of the calibrated model, $\sum_{i}L_{v_{i}}^{*}(\Theta ,\sigma )$, $v_{i}\in \{HCT,HR,SV,BP\}$, identified in Eq. 24 was reported.

#### 2) MODEL PREDICTIVE CAPABILITY PERFORMANCE

The validity of the model was examined based on its predictive capability performance. Two datasets were used to assess the predictive capability performance: sheep dataset (27 subjects) was used for an initial credibility assessment through a leave-one-out cross-validation procedure, while a subsequent validation was performed using independent swine test dataset (12 subjects). A virtual cohort generation tool developed in our prior research [8] was adapted and used with the mathematical model to generate virtual subjects for each individual.

#### 3) COMPARTMENT METHOD FOR VIRTUAL COHORT GENERATION

**FIGURE 2.**
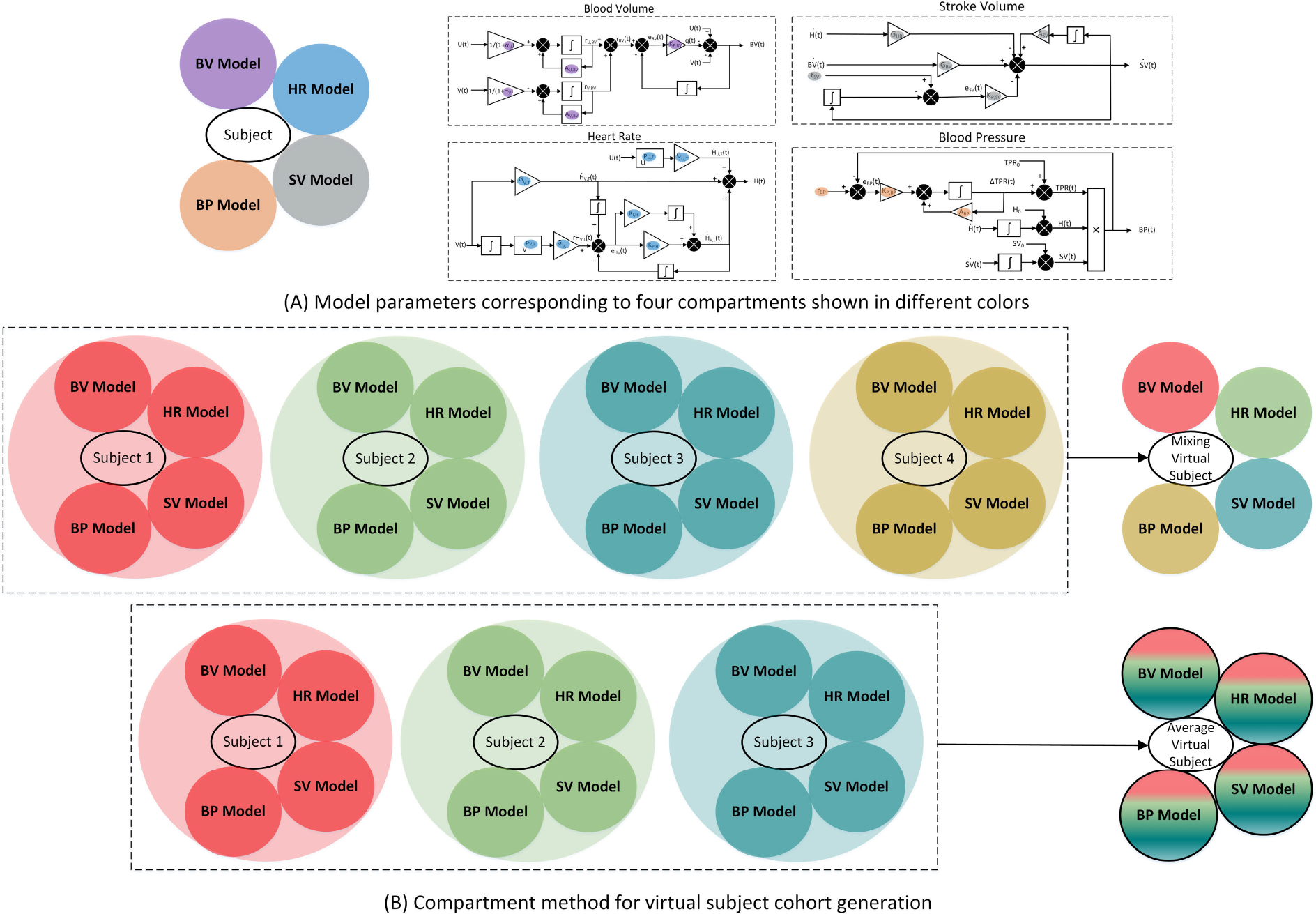
Virtual cohort generation using the compartment method. (A): All the parameters (except baseline parameters) corresponding to four compartments used for the virtual cohort generation. (B): A mixing virtual subject is created by combining compartments from four randomly selected subjects. An average virtual subject is created by averaging each compartment across 3 subjects.

To properly assess the predictive capability of the model, with an emphasis on the similarity of dynamic patterns exhibited by the simulated subjects with the test data, rather than similarity in their initial condition, we opted to sample the baseline parameters $\left(BV_{0},\;HCT_{0},\;HR_{0},\;SV_{0}\right.$, and $TPR_{0}$) from a normal distribution centered around their respective measured values in each test subject, with a standard deviation of 10%. This may adequately address the effects of measurement noise and inter-individual variation in individuals with a similar baseline hemodynamic state. The model parameters are divided into four compartments; BV, HR, SV, and BP, shown in Figure 2A. To generate virtual subjects, model parameters for each compartment were selected from one subject in the calibration dataset. This was done by selecting four subjects at random without repetition. For the leave-one-out cross-validation, 26 subjects were used, and 27 subjects were used for the secondary validation. Therefore, there were a total of 26^4^ = 456, 976 and 274 = 531, 441 possible combinations of four subjects (Figure 2B). The parameters for BV, HR, SV, and BP were obtained from each of the four randomly selected subjects, with one compartment selected from each subject. These simulated subjects are called mixing virtual subjects, which have a significant chance of being physiologically plausible, as the entire set of parameters associated with each compartment are obtained from a single calibration subject, instead of sampling individual parameters that may lead to non-physiological scenarios when combined. We also generated average virtual subjects by taking the average of model parameters of each compartment from three randomly selected subjects without repetition. This led to an additional 26^3^ = 17, 576 and 27^3^ = 19, 683 possible simulations for the leave-one-out and secondary validation, respectively (Figure 2B). Out of the possible simulated subjects, 474,552 and 551,124 for the cross-validation and secondary validation respectively, we selected 10,000 and 50,000 random subjects to investigate the impact of sample size on the prediction performance through the cohort generation.

#### 4) PREDICTIVE CAPABILITY ASSESSMENT

To ensure the credibility of our simulation results, we simulated 10,000 and 50,000 virtual subjects and filtered out any subjects with non-physiological responses. Specifically, we excluded subjects with HCT, HR, SV, and BP levels outside the range of 0–50%, 0–300 bpm, 0–100 ml, and 0–150 mmHg, respectively. Across the physiological simulations, we identified relevant subjects by selecting those with a similar pattern of hemodynamic response to the test subject. We quantified similarity using the NRMSE across HCT, HR, SV, CO, and BP, and we considered subjects with an NRMSE of 25% or less to be relevant. Finally, we constructed a “prediction envelope” for the relevant simulations by identifying the maximum and minimum simulated data points at each measurement time.

We tested the predictive capability of the model for each subject using a three-fold approach. First, we assessed the similarity between the most relevant simulated subject and the test data in terms of the maximum and average (across HCT, HR, SV, CO, and BP) NRMSE and NMAE, as well as -LL. Second, we measured the model’s predictive capability by calculating the average percentage of data points across HCT, HR, SV, CO, and BP that fall within the prediction envelope. We also determined the percentage of physiological and relevant simulations for each test subject. Finally, we reported the normalized interval score (NIS) [8] for HCT, HR, SV, CO, and BP. The NIS takes into account the width of the prediction envelope and applies a penalty if the measured data falls outside of it. A narrow prediction envelope results in a better, i.e., smaller, NIS score. The NIS score for measured variable v at time $t_{k}$ is computed by:

(27) $$\begin{array}{ll} & NIS_{v}\left(t_{k}\right)\\ & \;=\left\{\begin{array}{ll} \frac{\left(v_{u}\left(t_{k}\right)-v_{l}\left(t_{k}\right)\right)+\frac{2}{\alpha }\left(v_{l}\left(t_{k}\right)-v\left(t_{k}\right)\right)}{v\left(t_{k}\right)} & v\left(t_{k}\right)<v_{l}\left(t_{k}\right)\\ \frac{\left(v_{u}\left(t_{k}\right)-v_{l}\left(t_{k}\right)\right)+\frac{2}{\alpha }\left(v\left(t_{k}\right)-v_{u}\left(t_{k}\right)\right)}{v\left(t_{k}\right)} & v_{u}\left(t_{k}\right)<v\left(t_{k}\right)\\ \frac{v_{u}\left(t_{k}\right)-v_{l}\left(t_{k}\right)}{v\left(t_{k}\right)} & \mathrm{Else} \end{array}\right. \end{array}$$

where $v_{u}$ and $v_{l}$ are the upper and lower bound of the predictions envelope associated with variable v. The significance level α is considered to be 0.05 in this work.

The software code in the Appendix conducts a model credibility assessment by generating virtual subjects against a representative sheep subject. It then presents the prediction envelope and the best simulated subject based on the least NRMSE.

## III. RESULTS

In this section, we present the results for both calibration and predictive capability performance of the mathematical model to determine its reliability and effectiveness in accurately mimicing physiological variables and their dynamical changes during fluid perturbation.

### A. MODEL CALIBRATION

Table 4 presents all 25 mathematical model parameters identified for 27 subjects.

#### 1) CALIBRATION PERFORMANCE

**FIGURE 3.**
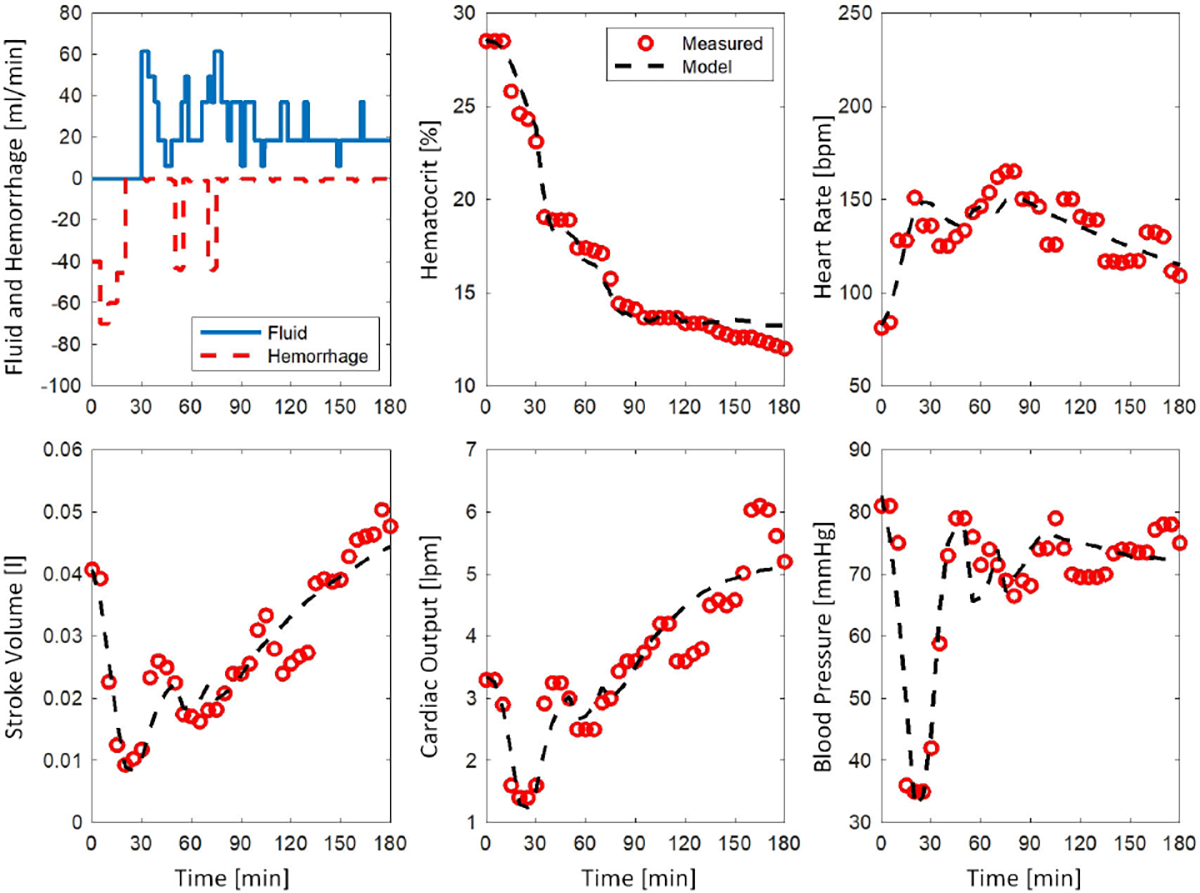
A representative example for the mathematical model calibration using sheep data. Mathematical model simulated response is shown in black.

Figure 3 shows a representative example for the mathematical model calibration performance. Table 1 shows the mathematical model calibration performance averaged across all sheep subjects. The results are reported in terms of the median, Q2, as well as the first and third quartiles, Q1 and Q3. The NRMSE and NMAE were reported for each variable, and although CO was not included in the calibration process, its performance was consistent with other variables. The calibration performance of the mathematical model was deemed satisfactory as the median NRMSE and NMAE were less than 10%.

**FIGURE 4.**
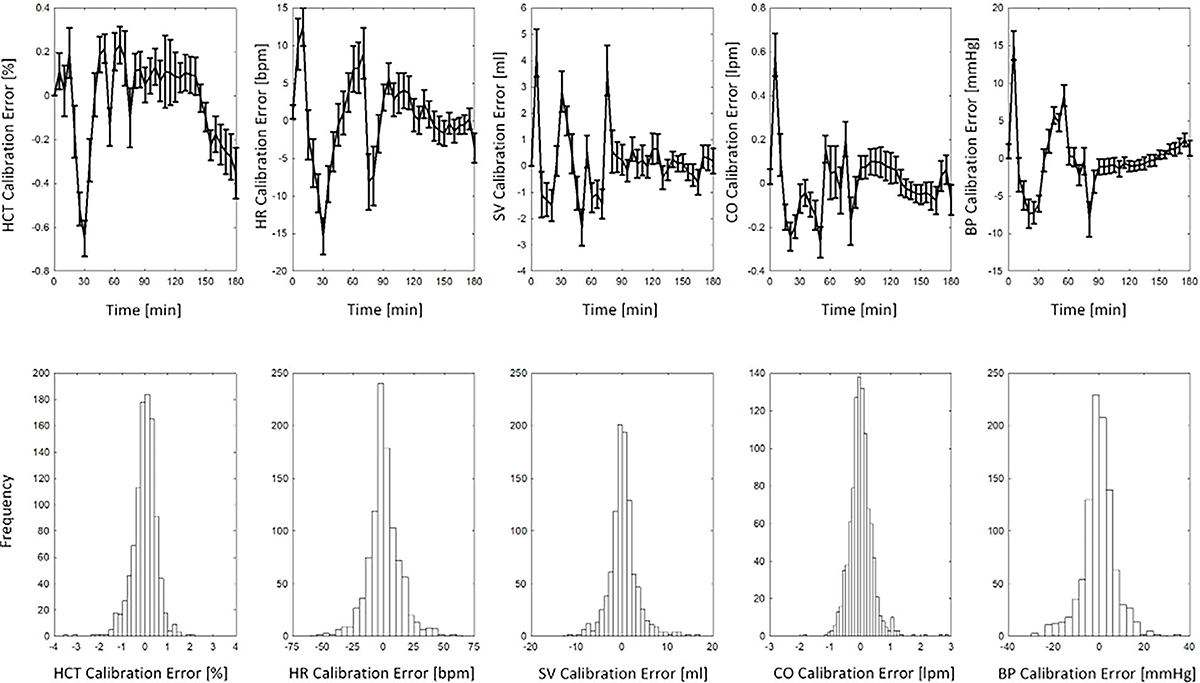
Mathematical model calibration error distribution. Top: distribution of error over time across sheep subjects. The standard error of the mean is depicted as a bar graph around the average error. Bottom: cumulative distribution of error across sheep subjects.

Figure 4 shows the distribution of errors across all subjects, both in terms of time and frequency, for each variable. Based on the distribution of error over time shown in the upper panel, errors are most likely to peak during the transient response to fluid perturbation. No discernible trend of error was observed over time, which suggests the absence of time bias in the mathematical model calibration process. The lower panel shows the cumulative distribution of error, which takes into account the frequency and magnitude of calibration errors across all the sheep subjects. The graph shows that errors are normally distrusted around zero for all variables, suggesting the absence of calibration bias in the mathematical model.

### B. MODEL VALIDATION

This section provides the results from the leave-one-out cross-validation using sheep data, as well as those from the subsequent validation using independent swine data.

#### 1) LEAVE-ONE-OUT CROSS-VALIDATION

**FIGURE 5.**
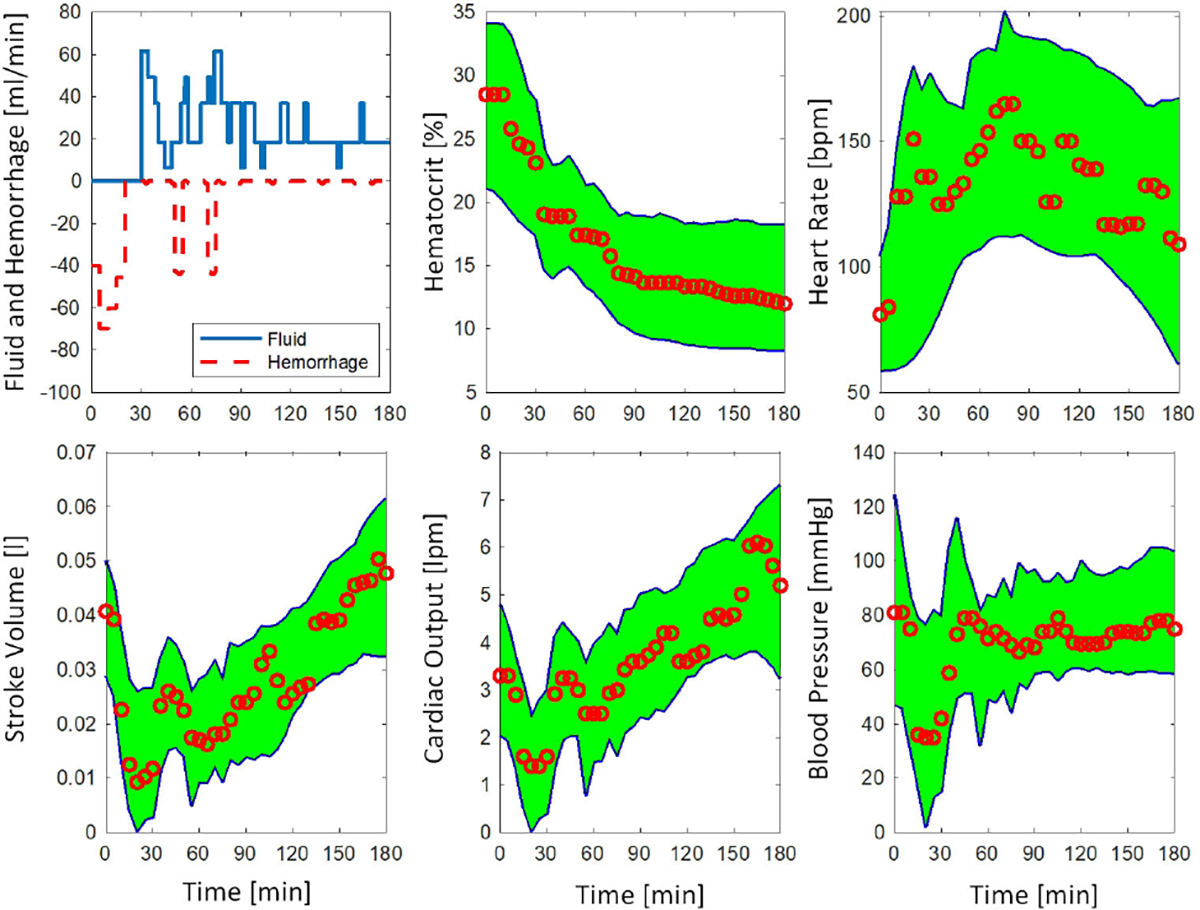
A representative example for the leave-one-out cross-validation using sheep data with 50,000 simulated subjects. The envelope covers 100% of the measured data. The relevant subjects with NRMSE<25% across all variables are included in the envelope.

Figure 5 shows a representative example for the leave-one-out cross-validation against the same test sheep subject as Figure 3. Table 2 shows results for the leave-one-out cross-validation. The results indicate that the virtual cohort with 50,000 sample size leads to statistically significantly better best virtual subjects in terms of NRMSE, NMAE, and -LL. It was also shown that 50,000-cohort had a significantly better data coverage. Lastly, the 10,000-cohort exhibited slightly lower NIS values for HR, SV, CO, and BP, but a statistically significant decrease in NIS was observed for HCT. The median NIS was acceptable in both 10,000 and 50,000 cohorts, where predicted responses deviated within ±50% of the corresponding observed variable (NIS<1 in all variables). The 10,000-cohort was not able to generate a prediction envelope for one sheep subject.

#### 2) VALIDATION VIA INDEPENDENT TEST DATA

**FIGURE 6.**
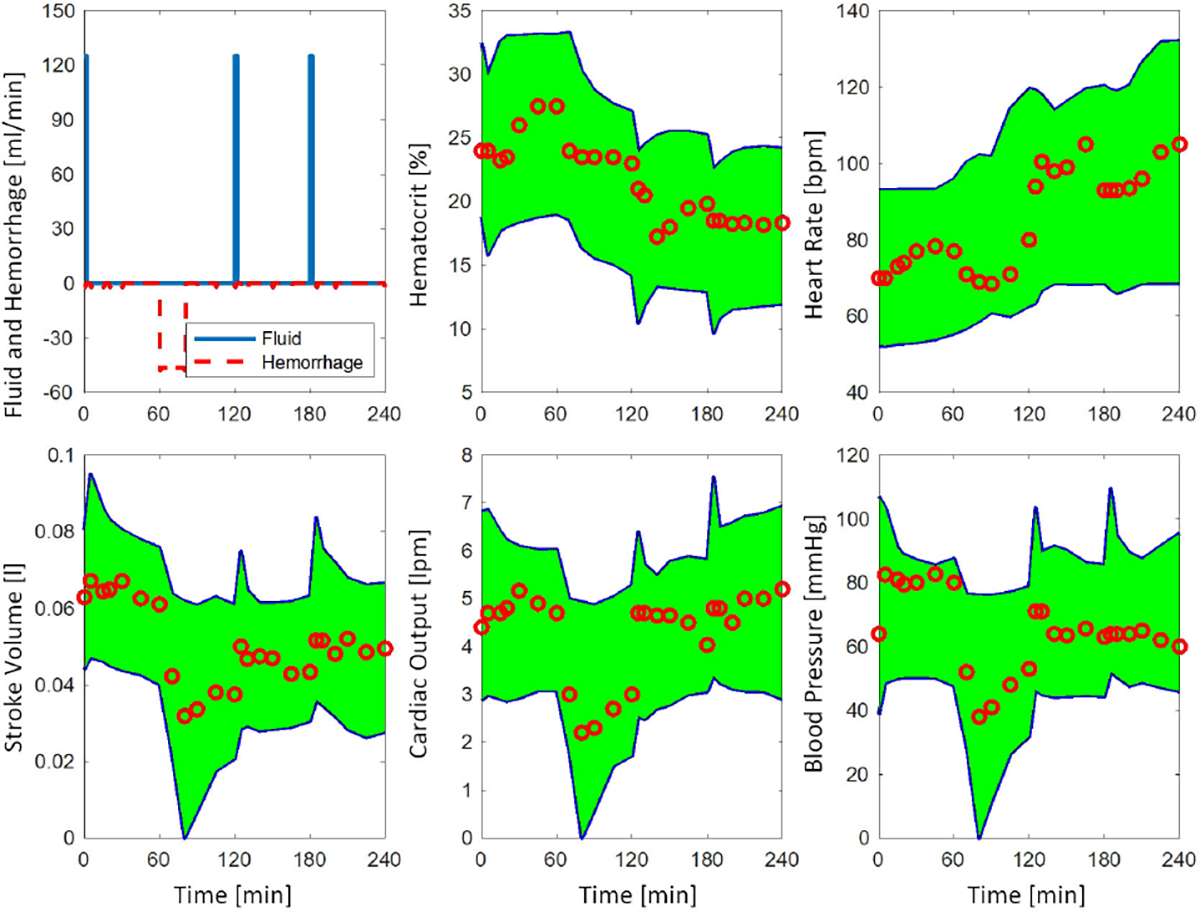
A representative example of the secondary validation using swine data with 50,000 simulated subjects. The envelope covers 100% of the measured data. The relevant subjects with NRMSE<25% across all variables are included in the envelope.

**FIGURE 7.**
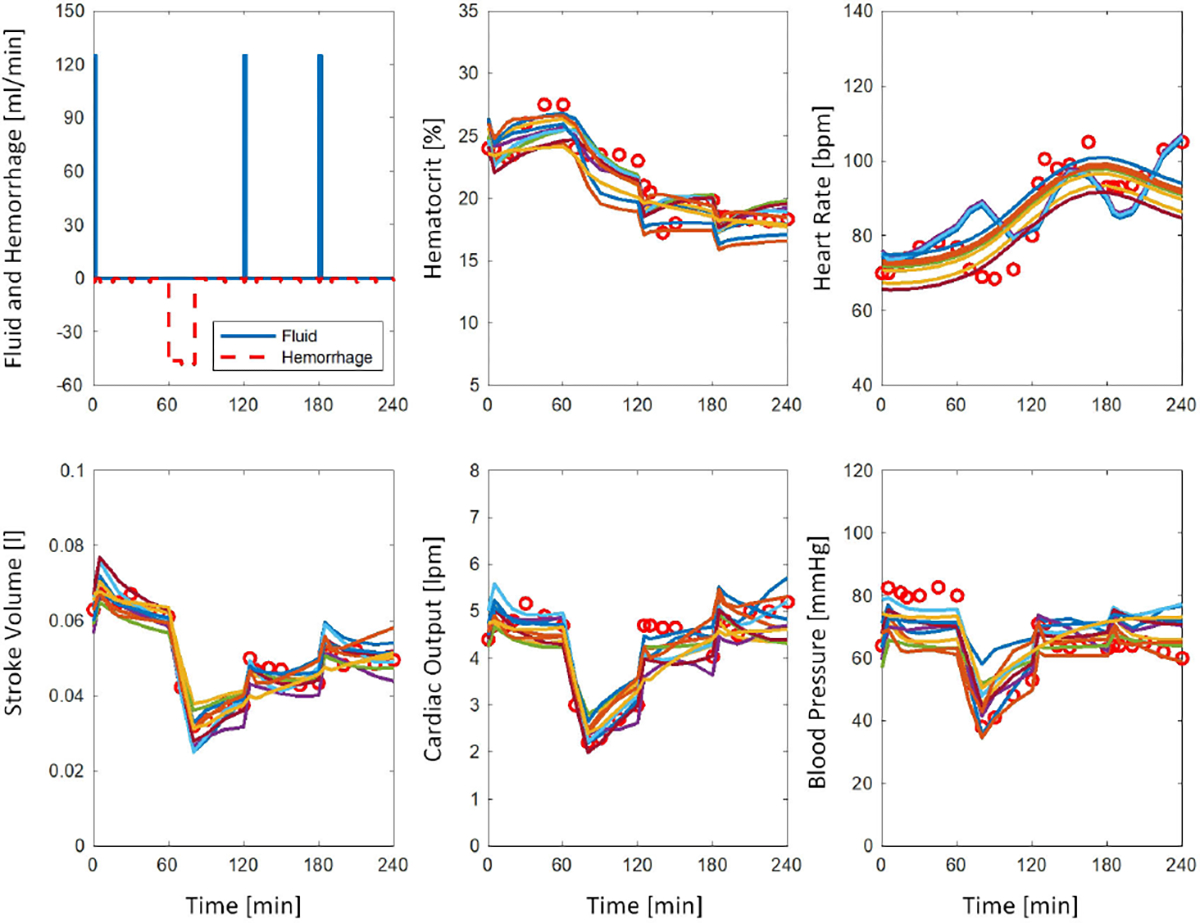
10 relevant virtual subjects with the least average NRMSE across HCT, HR, SV, CO, BP. Similar colors across panels represent different variables for the same subject. Red circles depict the measured values. The subject used is the same as in Fig. 6.

Figure 6 shows a representative swine data example with simulated 50,000 virtual subjects, where 100% of the data are covered by the green prediction envelope made by the relevant subjects. Figure 7 shows 10 relevant virtual subjects with the least average NRMSE for the subject shown in Figure 6. Table 3 includes the same information as Table 2, but for swine subjects used for the secondary validation. Results indicate significantly better best virtual subjects for the 50,000-cohort in terms of NRMSE, NMAE, and -LL (p<0.05). The median NIS was slightly smaller for the 10,000-cohort (p>0.1). The data coverage was also shown to be slightly better for the 50,000-cohort (p=0.08). The median NIS was <1, and deemed to be satisfactory in both 10,000 and 50,000 cohorts. Both cohorts with 10,000 and 50,000 samples were not able to generate a prediction envelop for one swine subject.

## IV. DISCUSSION

To address the critical need for in silico evaluation tools for PCLC medical technologies in fluid resuscitation, this research develops a mathematical model of the cardiovascular system and defines and employs rigorous methods for mathematical model validation. Although parsimonious, the developed mathematical model includes essential physiological mechanisms and variables that are involved during fluid perturbation. A compatible virtual cohort generation tool is defined that can effectively predict physiological responses under different infusion protocols and boundary conditions. A range of tools are also proposed for mathematical model credibility assessment. It is worth noting that the techniques employed for virtual cohort generation and mathematical model validation will have broader applicability beyond the specific cardiovascular mathematical model developed in this research. This section will delve into different aspects of the mathematical model development and validation process in detail.

### A. MODEL COMPLEXITY

The mathematical model accurately represents essential physiological responses to fluid perturbation, including changes in BV, HR, SV, CO, and BP. The mathematical model achieves this by incorporating the fundamental physiological mechanisms that underlie fluid perturbation, such as fluid shift, inotropic and vasoactive effects. A lumped-parameter mathematical modeling approach is employed to effectively reproduce these mechanisms while maintaining the model’s parsimony. We note that a mathematical model designed to generate a virtual patient cohort should ideally be parsimonious. This is because it can be challenging to sample a large number of mathematical model parameters across their physiological range through a reasonable number of simulations. When a mathematical model has a large number of parameters, the combination of sampled parameters can easily lead to non-physiological scenarios. As a result, the generated patients may not adequately represent a diverse range of normal and worst-case conditions, which can lower the chances of an effective PCLC evaluation. The mathematical model consists of a total of 25 parameters, out of which 5 are baseline parameters. These baseline parameters are sampled around the initial condition of the test subject. As a result, the model retains only 20 parameters to be sampled using a virtual cohort generation tool. It is worth noting that numerous existing mathematical models of the cardiovascular system comprise hundreds and thousands of parameters, posing a significant challenge for effective parameter sampling to generate patient cohorts (e.g., [21], [22], [23]).

### B. PHYSIOLOGICAL PLAUSIBILITY

The mathematical model is physiologically plausible and designed to capture the cardiovascular response to fluid perturbation based on fundamental physiological concepts. In particular, the BV mathematical model is built based on the physiological fluid shift mechanism that occurs when fluid is redistributed between the intravascular and interstitial spaces in response to changes in fluid balance [21]. The HR mathematical model is designed to capture the dynamic changes that occur in response to hemorrhage followed by fluid infusion, taking into account the complex interplay between the autonomic nervous system and cardiovascular response [8]. The SV mathematical model is constructed around the principles of the Frank-Starling mechanism [32], which elucidates the relationship between SV and HR. According to this mechanism, an elevated heart rate results in a shortened duration for cardiac filling during each cardiac cycle, leading to a reduction in stroke volume. The Frank-Starling mechanism also states that a reduction in BV results in decreased preload, the stretch of the ventricles before contraction. This diminished preload lowers the force of ventricular contraction, leading to a decrease in SV (opposite during fluid resuscitation). Therefore, the SV mathematical model incorporates the interplay between SV and both HR and BV, capturing the influence of these factors on cardiac performance. Lastly, the BP mathematical model is designed to replicate the vasoactive effect of fluid perturbations on TPR. This vasoactive effect is primarily induced by the sympathetic nervous system, leading to vasoconstriction under hemorrhage and vasodilation under fluid infusion [33]. Vasoconstriction narrows the blood vessels and increases systemic vascular resistance (SVR), while vasodilation expands the blood vessels and decreases SVR. The BP mathematical model incorporates a feedback-control mechanism to effectively simulate the vasoconstriction observed during fluid perturbations.

### C. MODEL CALIBRATION

The median values of NRMSE and NMAE among the sheep subjects were found to be below 10%, suggesting a satisfactory level of calibration performance. The calibration performance was the best for HCT, primarily due to the consistent physiological downward trend observed in HCT during fluid perturbations, coupled with the absence of significant challenging variability. This characteristic made it relatively easier to accurately replicate the HCT measurements, resulting in a superior calibration performance. The remaining variables, including HR, SV, and BP, exhibited consistent and still small errors. Although CO was not explicitly calibrated, the error associated with CO demonstrated a consistent pattern similar to that of HR and SV. This finding suggests that the calibration process was unbiased and not favoring any specific variable. Upon analyzing the distribution of error over time (Fig. 4, upper panel), it was observed that the largest calibration error occurred during the transient response to hemorrhage and infusion, whereas it diminished to relatively smaller values during the steady state. Notably, the error for HCT exhibited a decreasing trend after 120 minutes, likely due to the absence of a steady state response, as HCT generally follows a downward pattern during fluid infusion. For the remaining variables, the calibration error remained small and centered around zero after 100 minutes. Furthermore, when considering the frequency of error across all subjects, a normal distribution around zero was evident for all variables (Fig. 4, lower panel). The overall calibration performance displayed a balanced and satisfactory level of accuracy across all measured variables.

### D. VIRTUAL COHORT GENERATION

To effectively generate virtual subjects that accurately mimic realistic physiological scenarios, it is crucial to sample the combination of parameters instead of relying on independent sampling of each parameter. Our earlier research [8] demonstrated that an independent sampling of mathematical model parameters does not lead to an effective generation of simulated subjects. Specifically, cohorts with individually sampled parameters can generate numerous non-physiological simulations and fail to accurately replicate normal and worst-case conditions. In light of this, a compartment-based virtual cohort generation tool was adapted from our previous work [8] to facilitate the sampling of parameter combinations and enhance the generation of virtual subjects with improved physiological accuracy. It was easily customized to sample five baseline parameters using an independent normal distribution sampling procedure, with an intention to eliminate the effects of initial conditions in mathematical model credibility assessment. The number of compartments can be selected based on the context of use of the mathematical model, the sample size of calibration data, and the balance between computation cost and accuracy. More specifically, increasing the number of compartments allows for a greater number of potential simulations and enhances the likelihood of getting more accurate simulated subjects. However, it is important to note that, with a larger number of compartments, a larger cohort sample size may be necessary to encompass both normal and worst-case conditions.

### E. PREDICTIVE CAPABILITY

The inclusion of swine data in the validation dataset is especially valuable due to the fact that it was collected under distinct physiological conditions and experimental protocol design, compared to the calibration data. Notably, the swine subjects were anesthetized, which differs from the conscious state of the sheep subjects. Therefore, the mathematical model’s performance was tested across diverse physiological conditions, enhancing its evidence of validity and generalizability.

During the validation process, there were a few instances where the mathematical model failed to generate relevant virtual subjects. Specifically, for one sheep subject out of the total 27 sheep subjects, no relevant virtual subjects were generated using the 10,000-cohort. Similarly, for one swine subject, no relevant subjects were simulated under both 10,000 and 50,000 cohorts. In the case of the sheep subject, the individual exhibited a rare response in CO, where an increased CO was observed between 90–130 minutes, while no such response was evident in the BP measurements. Since the definition of relevant subjects requires NRMSE<25% across all variables, a poor prediction performance in one variable can impact the overall relevancy of the simulation, regardless of the accuracy of other variables. However, it is worth noting that with the 50,000 cohort, four relevant subjects were successfully generated for the same subject, indicating an increased likelihood of successful simulations using a higher cohort sample size even under rare conditions. Regarding the swine subject, it is important to note that the subject experienced a consistent low BP (30 mmHg) during the final 120 minutes of the recorded data. This occurrence was primarily attributed to the subject being in an anesthetized state. Despite the CO returning to baseline level during this period, the mathematical model failed to generate relevant virtual subjects. The lack of relevant simulations for this particular subject is because of the absence of the observed BP response pattern in the conscious sheep subjects used for mathematical model calibration. Specifically, there are differences in physiological compensation between anesthetized versus conscious hemorrhage and fluid resuscitation. General anesthesia exerts profound effects on BP and HR, influencing preload, afterload, and contractility. Hemorrhage occurring during general anesthesia may manifest more pronounced effects, wherein a hemorrhage of 10 ml/kg, for instance, might resemble the physiological impact of a 15–20 ml/kg blood loss. On the other hand, the swine subject aligns with observations in humans experiencing a 10 ml/kg hemorrhage followed by a 30 ml/kg crystalloid fluid bolus [34], demonstrating a substantial decrease in afterload after hemorrhage and fluid bolus. The absence of accounting for general anesthesia may contribute to model deviations from actual true values in this subject.

The median maximum NRMSE of the best virtual subject across all variables consistently hovered around 15% in both sheep and swine datasets. This suggests that the best virtual subject closely approximates the characteristics of the test subjects, indicating satisfactory similarity. As expected, these metrics were higher than those reported for the calibration performance, owing to the significant intersubject variability in response to fluid perturbation.

It was observed that approximately 60% of the simulations in sheep subjects and 40% of the simulations in swine subjects exhibited physiological characteristics, by our definition of physiological ranges (see section II–D4). The lower percentage of physiological simulations in the swine subjects can be attributed to differences in fluid perturbation profiles as well as variations in their baseline physiological condition. The proportion of relevant subjects was also determined for both the 10,000 and 50,000 cohorts. In sheep subjects, the median percentage of relevant subjects exceeded 1.3%, while in swine subjects, it exceeded 1.9%. This clearly indicates that only a small fraction of the simulations produced relevant subjects, highlighting the difficulty of accurately simulating subjects with similar response patterns to the test subject. The higher median value observed in the swine data was attributed to a large number of relevant subjects found in a few specific subjects. However, upon examining the first quartile values, it becomes apparent that the number of relevant subjects in the challenging swine subjects was relatively smaller. The prediction envelope exhibited a coverage rate exceeding 96% for both sheep and swine data, indicating a satisfactory representation of inter- and intrasubject variability within both validation datasets. Lastly, the median NIS reported across all physiological variables was less than 0.9, implying a maximum deviation of 45% in the predicted responses compared to the observed data. This indicates an acceptable level of deviation, considering the substantial inter and intrasubject variability experienced during fluid perturbation (Tables 2 and 3).

### F. VIRTUAL COHORT GENERATOR SAMPLE SIZE

For the 10,000- and 50,000-cohorts, the median number of relevant simulations (with NRMSE<25% across all variables) in each test subject was found to be 151 and 660 for the sheep subjects, respectively (Table 2). Similarly, for the swine subjects, the median number of relevant simulations was 208 and 930 for the cohorts of 10,000 and 50,000 samples, respectively (Table 3). It was shown that the cohort with larger sample size of 50,000 showed a significantly better performance in generating best virtual subject in both sheep and swine subjects. In particular, all NRMSE, NMAE, and -LL for the best simulated subject turned out to be statistically significantly better for the cohort with 50,000 simulations. The proportion of physiological and relevant simulations did not exhibit a significant difference between the two cohorts, highlighting the robustness of the virtual cohort generator in generating random subject simulations. However, the coverage of the prediction envelope was statistically significantly better in the 50,000-cohort of the sheep data, while it did not show a significant difference in the swine subjects. This suggests that the 50,000-cohort envelope performs better in simulating worst-case conditions, as a lower sample size in a cohort may encounter difficulties in simulating similar physiological responses. Lastly, the NIS scores did not show a significant difference between the two cohorts. The median NIS values were generally smaller in the 10,000-cohort, primarily because of a narrower envelope that had not many data points outside of it (see first quartile data coverage in sheep, Table 2). However, when considering the third quartile NIS values for HR in sheep data and CO and BP in swine data, the larger NIS values in the 10,000-cohort were a result of the improved performance of the 50,000-envelope under rare and worst-case conditions.

Overall, the results substantiate the mathematical model’s credibility across a broad range of physiological conditions associated with acute hemorrhage. The mathematical model successfully handled various boundary conditions, fluid perturbation protocols, and physiological scenarios, displaying strong predictive capability performance in the majority of cases. However, there were a few instances where the mathematical model exhibited poor performance with no or limited relevant simulations, such as the anesthetized swine subject with a consistent low BP. Further evaluation and consideration should be given to including these challenging subjects in the calibration data to enhance the mathematical model’s predictive capability in future PCLC medical device assessments.

This research is currently ongoing with the aim of enhancing the mathematical model’s credibility by evaluating its predictive capability performance when used with PCLC medical devices. Our plan involves collecting PCLC performance characteristics while conducting tests with the mathematical model’s virtual subjects, and comparing those characteristics with the ones from animal subjects that underwent the same PCLC algorithm for fluid infusion. We anticipate that under similar physiological conditions, the PCLC characteristics, such as rise time and settling time, demonstrate a similar distribution to that observed in real subjects. By validating the mathematical model’s behavior in a closed-loop setting, we aim to assess the mathematical model’s reliability and applicability for effectively simulating and predicting responses to acute hemorrhage followed by fluid infusion.

## V. CONCLUSION

This research involved the development and validation of a mathematical model that captures the cardiovascular response to acute hemorrhage and fluid infusion. The mathematical model was constructed based on physiological first principles, and a comprehensive set of metrics and tools were created to assess its calibration and predictive capability. The results demonstrated the mathematical model’s effectiveness in replicating normal and worst-case conditions across various boundary conditions and fluid perturbation protocols. These metrics can be easily extended to other modeling domains. The validated cardiovascular mathematical model holds potential for preclinical evaluation of fluid resuscitation PCLCs.

## Figures and Tables

**TABLE 1.** Mathematical model calibration performance. NRMSE: normalized root-mean-squared error. NMAE: normalized mean absolute error. -LL: negative log-likelihood for the normal distribution of calibration error. The results are averaged across all calibration subjects. The results are reported in terms of the Q2[Q1,Q3].

-LL		NRMSE[%]	NMAE[%]

129[108,160]	HCT	2.56[2.06,3.32]	1.98[1.63,2.66]
	HR	8.84[6.70,11.2]	6.95[5.24,8.57]
	SV	9.46[6.95,11.6]	6.59[5.38,8.18]
	CO	9.70[7.91,10.9]	7.48[6.34,8.63]
	BP	8.76[7.43,12.5]	6.55[5.49,9.08]

**TABLE 2.** Mathematical model predictive capability performance for the leave-one-out cross-validation. NRMSE: normalized root-mean-square error. NMAE: normalized mean absolute error. -LL: negative log-likelihood for the normal distribution of calibration error. The results are reported in terms of the Q2[Q1,Q3]. Results that exhibit a statistically significant difference (paired t-test,*p*<0.05) between the 10,000 and 50,000 sample sizes are indicated with an asterisk (*).

		10,000 sample	50,000 sample

Best virtual subject	Maximum NRMSE	16.3[14.4,18.8]	15.0[13.4,17.8]*
	Average NRMSE	12.7[11.3,14.3]	11.6[10.6,13.1]*
	Maximum NMAE	13.9[11.2,14.5]	12.2[10.6,14.0]*
	Average NMAE	10.2[9.20,11.4]	9.50[8.35,10.3]*
	-LL	228[195,249]	205 [184,231]*

Virtual cohort coverage	% physiological subjects	59.9[50.5,65.3]	59.9[51.0,65.3]
	% relevant subjects	1.51 [0.52,2.52]	1.32[0.47,2.39]
	% data coverage	96.3[91.2,99.5]	98.4[94.4,100]*

Prediction envelope NIS	HCT	0.55[0.51,0.59]	0.59[0.56,0.64]*
	HR	0.70[0.63,1.01]	0.71[0.67,0.91]
	SV	0.81[0.73,0.85]	0.87[0.76,0.93]
	CO	0.76[0.70,0.83]	0.83[0.74,0.90]
	BP	0.69[0.64,0.82]	0.75[0.70,0.86]

**TABLE 3.** Model predictive capability performance for the independent swine dataset. NRMSE: normalized root-mean-square error. NMAE: normalized mean absolute error. -LL: negative log-likelihood for the normal distribution of calibration error averaged. The results are reported in terms of the Q2[Q1,Q3]. Results that exhibit a statistically significant difference (paired t-test, *p*<0.05) between the 10,000 and 50,000 sample sizes are indicated with an asterisk (*).

		10,000 sample	50,000 sample

Best virtual subject	Maximum NRMSE	14.8[11.1,21.3]	14.6[11.2,19.5]*
	Average NRMSE	11.9[8.7,15.3]	11.619.10,14.5]
	Maximum NMAE	12.6[9.60,16.0]	11.5[9.40,14.4]*
	Average NMAE	9.90[7.58,11.8]	9.30[7.35,11.3]*
	-LL	141[83,152]	130[79,143]*

Virtual cohort coverage	% physiological subjects	40.9[30.7,47.6]	39.7[31.0,47.7]
	% relevant subjects	2.08[0.42,3.67]	1.8610.28,3.51]
	% data coverage	97.8[87.9,100]	98.9192.8,100]

Prediction envelope NIS	HCT	0.48 [0.46,0.52]	0.54[0.51,0.56]
	HR	0.53[0.50,0.58]	0.54[0.53,0.58]
	SV	0.75[0.71,0.87]	0.8010.74,0.86]
	CO	0.75[0.69,1.17]	0.86[0.77,0.89]
	BP	0.70[0.63,1.74]	0.76[0.73,1.51]

**TABLE 4.** Model parameters calibrated to 27 sheep datasets. No statistically significant difference was observed between the BV model parameters of LR and HEX, as evidenced by the results of a two-sample T-test with unequal variance. The three parameters exhibiting significant differences between LR and HEX (highlighted by underlined *p* values) are related to the HR and SV models, but with relatively lower physiological significance. These differences may not necessarily reflect physiological discrepancies, while the small sample size of HEX may have contributed to these discrepancies.

	BV Model Parameters					HR Model Parameters							SV Model Parameters					BP Model Parameters			Baseline Model Parameters				
Subject	$A_{U,BV}$	$A_{V,BV}$	$a_{U}$	$a_{V}$	$K_{P,BV}$	$G_{U,T}$	$G_{V,T}$	$G_{V,L}$	$P_{U,T}$	$P_{V,L}$	$K_{P,H}$	$K_{I,H}$	$G_{BV}$	$G_{HR}$	$A_{SV}$	$r_{SV}$	$K_{P,SV}$	$A_{BP}$	$K_{P,BP}$	$r_{BP}$	${\mathrm{HCT}}_{0}$	${\mathrm{BV}}_{0}$	$H_{0}$	${\mathrm{SV}}_{0}$	${\mathrm{TPR}}_{0}$
LR1	−0.0978	−0.0069	0.3108	0.2193	0.3807	0.2760	63.9160	38.9270	0.0021	0.8518	0.0021	0.0019	0.0040	0.0007	−0.0039	0.0559	0.0034	−0.0041	0.0055	72.0940	21.6000	2.3380	62.9790	0.0559	21.8780
LR2	−0.1450	−0.0072	−0.8142	0.3547	0.3846	0.1064	0.0003	0.9570	0.0195	0.3578	0.0000	0.0009	0.0061	0.0008	−0.0000	0.0589	0.0116	−0.2405	0.1888	75.2050	23.9400	2.5723	78.4800	0.0427	23.5830
LR3	−0.7551	−0.0028	−0.1490	0.5893	0.1375	0.0106	0.0487	0.0712	0.1226	0.5355	0.0323	0.0165	0.0147	0.1296	−0.0013	0.0534	0.0246	−0.0244	0.0117	71.5920	31.7000	1.8895	77.8610	0.0534	24.8240
LR4	−0.5425	−0.0051	−0.3322	0.4104	0.1352	5.0298	7.6321	0.5671	0.5778	0.0000	0.0615	0.0098	0.0044	0.0032	−0.0000	0.0863	0.0249	−0.2796	0.1252	53.0330	28.8300	2.4884	52.4690	0.0572	23.0260
LR5	−0.5013	−0.0255	−0.4886	0.3221	0.0688	0.4415	34.0430	28.7940	0.0064	0.7681	0.1290	0.0368	0.0031	0.0005	−0.0042	0.0407	0.0041	−0.0155	0.0174	62.1920	28.5000	2.7221	82.2410	0.0407	24.6720
LR6	−0.6107	−0.0104	−0.3297	0.2495	0.0861	8.5914	19.7470	3.2933	0.6000	0.1969	0.0045	0.0014	0.0163	0.0003	−0.0000	0.0368	0.0186	−0.4590	0.1840	58.1140	22.4960	1.9374	101.2200	0.0198	23.3810
LR7	−0.0681	−0.0108	0.1302	0.1461	0.3919	10.8640	0.0064	5.0610	0.5914	0.2400	0.0086	0.0009	0.0239	0.0001	−0.0029	0.0324	0.0003	−0.3342	0.2014	73.2170	30.3000	2.9268	101.8300	0.0324	23.2380
LR8	−0.3915	−0.0023	−0.6154	0.4163	0.0394	0.0000	5.8500	8.1290	0.0026	0.4058	0.0167	0.0023	0.0158	0.0003	−0.0097	0.0372	0.0097	−0.1602	0.3587	70.1100	23.8500	2.4996	86.0000	0.0372	25.6250
LR9	−0.3996	−0.0039	−0.3846	0.2086	0.0736	27.4100	13.0470	5.3117	0.5911	0.2006	0.0000	0.0018	0.0216	0.0000	−0.0000	0.0186	0.0100	−0.0000	0.0016	92.0680	20.4000	2.5730	96.4900	0.0442	24.0750
LR10	−0.7050	−0.0095	−0.3070	0.4492	0.1310	16.6650	30.2970	4.4546	0.4740	0.1837	0.0018	0.0021	0.0252	0.0001	−0.0000	0.0363	0.0000	−0.3716	0.2477	70.4470	26.1000	2.1358	80.0040	0.0362	35.8620
LR11	−0.4477	−0.0353	−0.4845	−0.1170	0.3171	2.1241	85.8680	65.7530	0.3140	0.8472	0.0159	0.0185	0.0257	0.0004	−0.0006	0.0638	0.0006	−0.6096	0.2021	68.2090	21.4200	2.2085	58.0000	0.0638	21.0810
LR12	−0.8273	−0.0072	−0.0678	0.3589	0.2275	1.7087	0.0126	19.4120	0.3326	0.5834	0.0007	0.0011	0.0200	0.0002	−0.0011	0.0233	0.0662	−0.0135	0.0262	64.9880	27.1475	1.7498	91.0840	0.0227	31.2000
LR13	−0.8332	−0.0045	−0.0975	0.3986	0.3767	8.9324	23.4210	143.150	0.4994	0.9933	0.0122	0.0001	0.0000	0.0010	−0.0000	0.0241	0.1194	−0.7064	0.5237	70.5550	22.0000	1.9840	70.3100	0.0420	25.5170
LR14	−0.7081	−0.0031	−0.2577	0.9681	0.1062	15.0990	5.8894	10.4790	0.4896	0.3374	0.0000	0.0011	0.0451	0.0001	−0.0004	0.0641	0.0006	−0.5601	0.1440	73.7630	24.1756	2.7041	78.0600	0.0641	19.7990
LR15	−0.8669	−0.0060	−0.0015	0.2671	1.0134	0.0860	0.3959	11.7090	0.0576	0.4536	0.1433	0.3791	0.0003	0.0008	−0.0103	0.0507	0.0139	−0.0136	0.0117	54.1830	22.8191	1.6075	71.0230	0.0507	25.7690
LR16	−0.1341	−0.0045	−0.1310	0.6688	0.1417	0.9799	0.0046	1.2366	0.0002	0.0003	0.0000	0.0009	0.0411	0.0003	−0.0025	0.0635	0.0025	−0.5815	0.2559	84.6170	32.7114	2.7728	74.0000	0.0635	19.5740
LR17	−0.3874	−0.0146	−0.4408	0.4043	0.2636	2.9159	158.710	49.3460	0.1444	0.6857	0.0166	0.0021	0.0000	0.0004	−0.0000	0.0618	0.0009	−0.4371	0.0390	75.2130	18.5103	2.5353	76.0000	0.0618	16.0690
LR18	−0.0108	−0.0045	0.6689	0.8124	0.2068	47.6860	0.0058	4.5771	0.5274	0.0016	0.0031	0.0009	0.0111	0.0001	−0.0062	0.0153	0.0050	−0.0002	0.0393	59.4830	23.2163	1.5727	144.6900	0.0153	35.6520
LR19	−0.0192	−0.0217	0.2444	0.5654	0.8488	1.4826	64.9970	18.3250	0.0252	0.3302	0.0000	0.0007	0.0122	0.0001	−0.0032	0.0235	0.0005	−0.0613	0.0933	56.3170	24.4936	3.0070	50.6580	0.0235	36.3290
LR20	−0.6975	−0.0014	−0.2900	1.4417	0.1304	5.6959	30.4410	4.1683	0.4622	0.2455	0.0127	0.0013	0.0000	0.0009	−0.0061	0.0486	0.0053	−0.0195	0.0000	90.0000	23.6109	2.6526	77.7370	0.0486	23.2270
LR21	−0.0505	−0.0034	0.1805	0.5397	0.6838	1.9980	0.2040	3.9582	0.0074	0.0496	0.0001	0.0006	0.0205	0.0002	−0.0018	0.0383	0.0022	−0.0419	0.0019	84.0000	20.0870	2.1077	102.9700	0.0383	20.0860
LR22	−0.0564	−0.0038	0.2521	1.2610	0.9384	0.0000	0.0000	5.8679	0.0007	0.3803	0.0015	0.0019	0.0258	0.0000	−0.0000	0.0317	0.0097	−0.0005	0.0000	77.0000	28.0000	2.4154	89.4410	0.0565	13.8100
HEX1	−0.4134	−0.0108	−0.5858	0.0574	0.3597	2.8817	7.6022	0.1251	0.5052	0.0000	0.0000	0.0055	0.0086	0.0007	−0.0000	0.0479	0.0047	−0.1053	0.4848	82.5260	28.0600	2.3571	81.4080	0.0420	26.1400
HEX2	−0.0132	−0.0033	−0.0023	0.5381	0.2181	0.0826	0.0021	4.5615	0.0671	0.4849	0.0098	0.0035	0.0763	0.0005	−0.0000	0.0648	0.0049	−0.0176	0.0061	66.3600	32.1180	2.1296	51.8390	0.0635	33.0970
HEX3	−0.0089	−0.0188	0.2797	0.3800	0.5514	0.9835	16.7130	1.9736	0.0020	0.0007	0.0001	0.0010	0.0541	0.0004	−0.0000	0.0650	0.0000	−0.0093	0.0022	47.5560	26.5600	2.5882	62.8720	0.0650	20.1010
HEX4	−0.0475	−0.0179	−0.0857	0.0903	0.3710	0.9998	39.0210	28.3840	0.2056	0.7179	0.0008	0.0008	0.0316	0.0002	−0.0002	0.0425	0.0002	−0.5576	0.2294	69.2390	25.0090	2.3429	80.8320	0.0425	24.5480
HEX5	−0.3730	−0.5721	−0.6143	−0.4416	0.0129	2.6714	93.6290	5.0596	0.1272	0.1252	0.0353	0.0081	0.0285	0.0002	−0.0000	0.0380	0.0000	−0.0325	0.0118	44.3770	20.6590	1.9817	84.4890	0.0380	25.6670
																									
P-value	0.054	0.359	0.813	0.092	0.864	0.033	0.736	0.192	0.436	0.450	0.291	0.302	0.102	0.320	0.002	0.289	0.037	0.519	0.811	0.297	0.449	0.688	0.243	0.389	0.571

## APPENDIX

### SOFTWARE CODE

The software code for generating virtual subjects against a representative sheep subject to assess the model predictive capability performance can be found here: https://github.com/dbp-osel/Credible-mathematical-model-of-the-cardiovascular-system-response-to-fluid-perturbation

### CALIBRATED MODEL PARAMETERS

The calibrated model parameters for all 27 sheep subjects are presented in Table 4.
